# A Bayesian approach to analysing cortico-cortical associative stimulation induced increases in the excitability of corticospinal projections in humans

**DOI:** 10.1007/s00221-020-05943-3

**Published:** 2020-10-23

**Authors:** Richard G. Carson, Antonio Capozio, Emmet McNickle, Alexander T. Sack

**Affiliations:** 1 Trinity College Institute of Neuroscience and School of Psychology, Trinity College Dublin, Dublin, Ireland; 2 School of Psychology, Queen’s University Belfast, Belfast, Northern Ireland, UK; 3 School of Biomedical Sciences, Faculty of Biological Sciences, University of Leeds, Leeds, England, UK; 4 Department of Cognitive Neuroscience, Faculty of Psychology and Neuroscience, Maastricht University, Maastricht, The Netherlands

**Keywords:** Bayes factors, Paired associative stimulation, Corticospinal, Motor evoked potentials, Plasticity, Arm

## Abstract

Repeated pairing of transcranial magnetic stimulation (TMS) over left and right primary motor cortex (M1), at intensities sufficient to generate descending volleys, produces sustained increases in corticospinal excitability. In other paired associative stimulation (PAS) protocols, in which peripheral afferent stimulation is the first element, changes in corticospinal excitability achieved when the second stimulus consists of brief bursts of transcranial alternating current stimulation (tACS), are comparable to those obtained if TMS is used instead (McNickle and Carson 2015). The present aim was to determine whether associative effects are induced when the first stimulus of a cortico-cortical pair is tACS, or alternatively *subthreshold* TMS. Bursts of tACS (500 ms; 140 Hz; 1 mA) were associated (180 stimulus pairs) with single magnetic stimuli (120% resting motor threshold rMT) delivered over the opposite (left) M1. The tACS ended 6 ms prior to the TMS. In a separate condition, TMS (55% rMT) was delivered to right M1 6 ms before (120% rMT) TMS was applied over left M1. In a sham condition, TMS (120% rMT) was delivered to left M1 only. The limitations of null hypothesis significance testing are well documented. We therefore employed Bayes factors to assess evidence in support of experimental hypotheses—defined precisely in terms of predicted effect sizes, that these two novel variants of PAS increase corticospinal excitability. Although both interventions induced sustained (~ 20–30 min) increases in corticospinal excitability, the evidence in support of the experimental hypotheses (over specified alternatives) was generally greater for the paired TMS-TMS than the tACS-TMS conditions.

## Introduction

In the period since the first utilisation of the technique, paired associative stimulation (PAS) has become a method of choice with which to investigate the expression of neural plasticity at a systems level in humans (Carson and Kennedy 2013). The appeal arises, at least in part, from a resemblance to certain facets of spike-timing dependent plasticity (STDP, e.g., Müller-Dahlhaus et al. 2010). In particular, it has been observed that: (1) the polarity of the changes in corticospinal excitability induced by PAS are contingent upon the order in which stimulation is delivered to the (presumed) pre- and post-synaptic targets, and (2) that to be effective the ISIs must lie within a restricted (milliseconds) range (Wolters et al. 2003). These observations notwithstanding, qualitatively equivalent outcomes (i.e., suggestive of associative plasticity) have been achieved using methods that deviate from the notional requirements for STDP—for example using stimulus pairs that cannot be defined in terms of a discrete ISI (e.g.,Ridding and Taylor 2001; Carson et al. 2013; McNickle and Carson 2015; Shulga et al. 2016; Carson and Rankin 2018; Tolmacheva et al. 2019).

In assessing the effects of PAS on corticospinal excitability, as for many other forms of non-invasive brain stimulation (NIBS), it has been customary to rely upon null hypothesis significance testing (NHST). This approach is used to establish whether, on the basis of the observed data, the null-hypothesis of no effect can be rejected. The limitations of this approach have been outlined exhaustively (e.g.,Wagenmakers 2007; Greenland et al. 2016). Detailed description of these deficiencies is beyond the scope of the present article. Suffice to say, it has been shown that NHST can increase publication bias (Ioannidis 2005; van Assen et al. 2014), and the prevalence of undesirable research practices (Masicampo and Lalande 2012; O’Boyle et al. 2016; Perneger and Combescure 2017). The use of Bayesian inference in general, and the adoption of Bayes factors in particular, has been advocated widely as a means of dealing, at least in part, with the problems of NHST (e.g., Dienes 2016). In contrast to the significant/non-significant dichotomy of NHST, Bayes factors yield evidence concerning each of the hypotheses under consideration. A specific advantage of this approach is that models and parameters that are predictive of empirical data receive a boost in credibility. Models and parameters that exhibit poor levels of prediction suffer a consequential decline in plausibility (Wagenmakers et al. 2016). It has been proposed previously (de Graaf and Sack 2018) that with respect to NIBS, Bayesian methods may be used to help formalize the inferences drawn on the basis of null results. In the present study, we take the further step of using Bayes factors to assess the evidence in support of specific experimental hypotheses—concerning two types of PAS, which are defined quantitatively in terms of predicted effect sizes.

We evaluated two new variants of an associative protocol involving stimulation of interconnected brain areas (Rizzo et al. 2009, 2011; Koganemaru et al. 2009), in which pairs of magnetic stimuli at intensities sufficient to generate descending corticospinal volleys (i.e., in extant studies) were delivered over left and right M1. In the first new variant, the intensity of the first stimulus was lower than that used previously—55% of resting motor threshold (rMT), and the ISI set to 6 ms. It has been shown that these parameters can potentiate the amplitude of the motor evoked potential (MEP) elicited by the second stimulus of the pair (Bäumer et al. 2006). In the second new variant, 500 ms of transcranial alternating current stimulation (tACS) at 140 Hz was used instead as the first stimulus of the associated pair. Quantitative hypotheses expressing the degree to which these variants would induce increases in corticospinal excitability were formulated using effect size estimates derived from previous research.

## Material and methods

### Participants

Fourteen healthy volunteers (nine females, mean age = 24, s.d = 2.58; and five males, mean age = 25, s.d = 1.54) participated in the study. All were right handed according to the Edinburgh handedness inventory (Oldfield 1971), and gave informed consent to procedures approved by the relevant Trinity College Dublin Ethics Committee, which (with the exception of study pre-registration) were conducted in accordance with the Declaration of Helsinki. The sample size was guided by previous experiments in which tACS-based associative stimulation protocols were employed (McNickle and Carson 2015). In the language of Bayesian analysis, our stopping rule was to run as many participants as is “traditional” in the field (Dienes 2008). It was our original intent to recruit and test 16 participants. Due to circumstances unrelated to the testing protocol, one person was able to complete only a single session. Another participant exhibited consistently high levels of background EMG that precluded their inclusion. The order of allocation to conditions was partially counterbalanced across participants. In line with current recommendations (Nitsche et al. 2008), successive testing sessions were separated by at least 7 days. For each participant, all sessions commenced at the same time of day—to control for any potential influence of circadian rhythms (Sale et al. 2007). None of the participants had previous experience of transcranial alternating or direct current stimulation.

### Recording and stimulation procedures

The participants were seated with the upper limbs supported and stabilized by vacuum cushions, the forearms in mid-pronation and the elbows semi-flexed (100–120˚). Electromyographic (EMG) activity was recorded from the right flexor carpi radialis (FCR) and extensor carpi radialis brevis (ECR) muscles (Riek et al. 2000), using pairs of silver chloride (AgCl) electrodes. EMG signals were amplified (gain = 1000), bandpass filtered (30–1000 Hz), and digitized at a sampling rate of 4 kHz.

Magnetic stimuli were delivered to the left primary motor cortex (M1) by a Magstim 200 stimulator (Magstim Company, Whitland, Dyfed, UK), using a figure of eight coil (internal wing diameter 70 mm), located at the optimal position (“hot spot”) to obtain a motor evoked potential (MEP) in the FCR muscle of the contralateral (right) arm. The coil was placed so that the axis of intersection between the two loops was oriented at approximately 45 degrees to the sagittal plane, to induce posterior to anterior current flow across the motor strip. Once the hot spot was established, the lowest stimulation intensity at which MEPs with peak-to-peak amplitude of approximately 50 µV were evoked in at least five of ten consecutive trials was taken as resting motor threshold (rMT). In the paired TMS-TMS condition, the first magnetic stimulus was delivered at the “hot spot” overlying the right primary motor cortex via a separate coil (internal wing diameter 60 mm) and Magstim 200 stimulator. This coil was also oriented at approximately 45 degrees to the sagittal plane, to induce posterior to anterior current flow. The rMT for the left FCR was then established independently, as described above. Markings to aid in guidance of the coils were made directly on the scalp. The coils were supported by means of clamps. The positions and orientations of the coils was monitored continuously, and if necessary adjusted to align with the scalp markings.

**Fig. 1:**
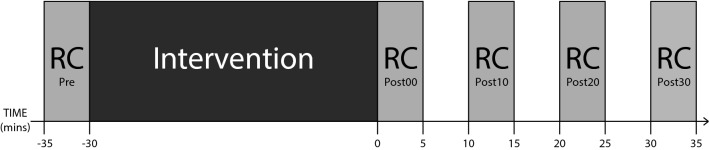
The time course of each experimental session is represented schematically. A recruitment curve (RC) was measured at the beginning of testing (‘Pre’) followed by a 30 min intervention that differed according to the experimental condition. Further recruitment curves were measured immediately following (‘Post00′) and at 10 (‘Post10′), 20 (‘Post20′) and 30 (‘Post30′) minutes following the completion of the intervention

Prior to each intervention (Pre), a MEP recruitment curve was obtained by delivering TMS over the left hemisphere at 10% increments of intensity between 90 and 160% of the rMT. Six stimuli were delivered at each level of intensity. A further 12 stimuli were delivered at 120% rMT. The order of delivery was randomised. The interval between successive stimuli was 10 s on average—“jittered” by 50%, such that the range was between 5 and 15 s. The total duration of the sequence was approximately 5 minutes. Average MEP amplitudes obtained at 90% and 100% rMT were calculated to ensure that the threshold had been correctly determined. In cases where the averaged MEPs for these intensities did not correspond to the expected values (i.e. < 50 μV at 90% rMT, 50-100 μV at 100% rMT), the threshold intensity was adjusted accordingly and another recruitment curve was obtained. Equivalent sets of stimuli (without adjustments of threshold) were delivered immediately following the intervention (Post0) and at 10 (Post10), 20 (Post20) and 30 (Post30) minutes thereafter (Fig. 1). The first of these sets (i.e., Post0) always commenced within 30 s following completion of the intervention. There was a break of 5 minutes after the delivery of each such set of stimuli prior to commencement of the subsequent set.

#### Transcranial alternating current stimulation (tACS)

Flexible electrode paddles were placed within two saline-soaked 5 cm x 5 cm sponges and fixed securely on the scalp using non-conducting elastic straps. One electrode was placed over right M1 at the FCR “hot spot” determined previously by TMS. The other electrode was placed over the contralateral supraorbital area. A battery-driven stimulator (AM Systems Model 2200, U.S.A.) controlled by Signal software (Cambridge Electronic Design, Cambridge, UK.) was used to deliver 500 ms duration bursts of bipolar sinusoidal alternating current at a frequency of 140 Hz and an amplitude of 1 mA. The current density was 0.04 mA/cm^2^. Electrode impedance was monitored and maintained below 5kΩ.

#### Interventions

**Fig. 2:**
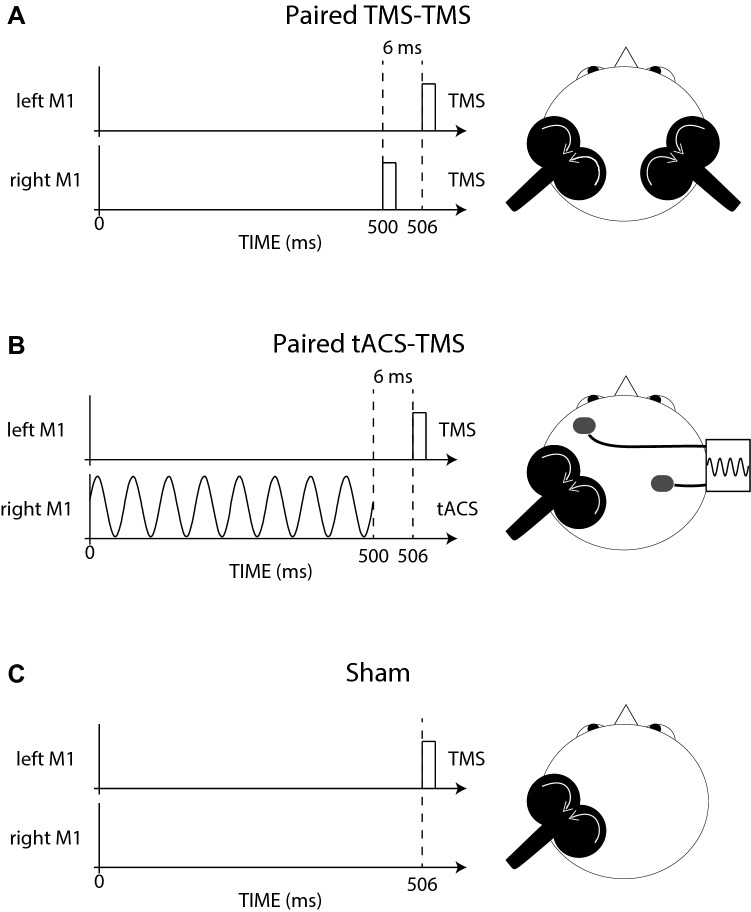
The three interventions are represented schematically. In each case, 180 TMS pulses were applied over the left primary motor cortex (M1) at an intensity of 120% resting motor threshold (rMT). In the paired TMS-TMS condition (**a**), TMS was delivered to the right M1 at an intensity of 55% rMT, 6 ms prior to each of the 180 magnetic stimuli delivered to the left M1. In the paired tACS-TMS condition (**b**), 500 ms duration trains of tACS were applied between an electrode placed over right M1 and a contralateral supraorbital electrode, at a frequency of 140 Hz and amplitude of 1 mA. The train commenced 506 ms prior to, and concluded 6 ms prior to, each of the 180 magnetic stimuli. In the Sham condition (**c**), no stimulation was applied in addition to the TMS delivered to the left M1. The white arrows superimposed on the images of the magnetic stimulating coils represent the direction of current flow in the coil

Sham condition. The electrode paddles were placed on the scalp in the manner described above. Following the completion of (Pre) MEP recruitment curve procedure, direct current stimulation (tDCS) was applied such that it increased linearly from 0 to 1 mA over a period of 10 seconds, and then decreased to 0 mA during the 10 seconds following. The participants perceived the tDCS as a weak tingling sensation localised to the scalp. Thereafter, 180 TMS pulses were applied over the left M1 at an intensity of 120% rMT (Fig. 2a). The interval between successive pulses was 10 s on average (~ 0.1 Hz)—“jittered” by 50%, such that the range was between 5 and 15 s.

#### Paired TMS-TMS condition

A total of 180 magnetic stimuli were delivered over the left M1 at an intensity of 120% rMT. The interval between successive stimuli was 10 s on average (~ 0.1 Hz)—“jittered” by 50%, such that the range was between 5 and 15 s. Six (6) ms prior to each such stimulus, TMS was delivered to the right M1 at an intensity of 55% rMT (Fig. 2b).

#### Paired tACS-TMS condition

Magnetic stimuli were delivered over the left M1 in the manner described for the paired TMS-TMS condition. A 500 ms duration train of tACS applied between the electrode placed over right M1 and the contralateral supraorbital electrode, at a frequency of 140 Hz and an amplitude of 1 mA (zero centred, i.e., 0.5 mA zero-to-peak/trough), commenced 506 ms prior to, and concluded 6 ms prior to, each of the 180 magnetic stimuli applied to left M1 (Fig. 2c).

In all conditions, the duration of the intervention was 30 min.

### Data analysis

The root mean square (rms) of the background EMG recorded in FCR and ECR was calculated for a window 93 ms to 3 ms before TMS onset. If the value was greater than 2.5 μV for either muscle, the corresponding MEP was disregarded. As a further means of eliminating instances in which elevated excitability of the spinal motoneuron pool may have influenced the MEP amplitude, we first calculated for each participant (separately for FCR and ECR) the quartiles for all background rms EMG values retained following the screening procedure described above. In the event that an individual rms value was above the upper whisker of the distribution (in this instance set to the third quartile plus 1.5 times the interquartile range) the corresponding MEP was disregarded. Overall, 86.4% of the responses were retained (Pre–87.2%, Post0–86.7%, Post10–83.4%, Post20–89.2% and Post30–85.5%). Following Cavaleri et al. (2017), it was a further requirement that a minimum of five qualifying MEP amplitude measurements were present for every analysis cell (i.e., for every stimulation intensity for every person in every condition).

For the retained recordings, the mean (peak-to-peak) of the natural logarithm transformed amplitude of the MEPs elicited at the eight respective stimulation intensities was calculated (Nielsen 1996). The values thus obtained were then inverse transformed to SI units (i.e., mV). For each time of measurement (Pre, Post0, Post10, Post20 and Post30), the summated area under the recruitment curve (AURC)—bounded by magnetic stimulation intensity and MEP amplitude (in units of mV.T), was obtained using the trapezoidal rule. It has been demonstrated elsewhere (Carson et al. 2013) that the AURC is a reliable measure of the state of corticospinal projections to hand and forearm muscles, which has construct, face, and concurrent validity.

The normality of the distribution of AURC values obtained in each analysis cell (i.e., separately for each condition) was assessed using the Shapiro-Wilks test. On the basis of these analyses it could not be inferred that any distribution of AURC values deviated from normality to a reliable degree (Sham: *p* = 0.05–0.94; tACS-TMS: *p* = 0.31–0.92; TMS-TMS: *p* = 0.19–0.36). As a consequence of the stringent steps that were applied to eliminate the influence of MEPs generated in the context of elevated spinal motoneuron excitability (described above), there were a small number of cells within the analysis design for which an AURC measure was not available (i.e. for a single participant at a single time of measurement). There were 2 such values (of 70) in the sham condition, 2 (of 70) in the tACS-TMS condition, and 2 (of 70) in the TMS-TMS condition. For all such instances (1 at Post0, 2 at Post10, 1 at Post20, 2 at Post30) no participant accounted for more than one such value. As the Bayesian analysis methods applied in the present study do not deal easily with such “missing values”, these were imputed using a sequential nearest neighbour imputation (kNN) method (using a *k* value of 10), implemented via the VIM package in R (Templ et al. 2011, 2012). This is a non-parametric method, that matches a (missing) point with its closest k neighbours in a multi-dimensional space defined by relevant variables. Replacements for missing values are extracted from cases (donors) that are similar to the recipient with respect to observed characteristics—in this case: the participant, the condition, and the time point. The assumption is that the missing value can be approximated by the values of the points closest to it.

The generation of Bayes Factors was conducted separately for each condition, and each time of measurement contrast (i.e., pre versus post) using the BayesFactor package in R (Rouder et al. 2009). In all cases, an informed prior was used. The point estimate of the prior (Table 1) was based on effect size estimates (Cohen’s d) derived from data given in Rizzo et al. (2009), for contrasts between measurements obtained before the intervention (Pre), and at each time point following the intervention (Post0, Post10, Post20, Post30). The values shown for the Post10 and Post20 contrasts were interpolated (linearly) on the basis of the test statistics reported for Post0 and Post30 in the Rizzo et al. (2009) paper. The lower and upper boundaries of the priors defined on the basis of these effect size estimates (Table 1) were calculated using the 95% confidence intervals for the effect sizes given in McNickle and Carson (2015) (i.e., corresponding to contrasts between Pre and Post0, Post10, Post20, and Post30, respectively).

**Table 1:** Effect size estimates (Cohen’s d) derived from data given in Rizzo et al. (2009), for contrasts between measurements obtained prior to the intervention (Pre), and at each time point following the intervention (Post0, Post10, Post20, Post30)

	Effect size (d)	Lower	Upper
Contrast			
Pre vs. Post00	0.601	0.403	0.799
Pre vs. Post10	0.684	0.474	0.893
Pre vs. Post20	0.766	0.556	0.976
Pre vs. Post30	0.849	0.634	1.063

The values shown for the Post10 and Post20 contrasts were interpolated (linearly) on the basis of the test statistics reported for Post0 and Post30 in the Rizzo et al. (2009) paper. The lower and upper boundaries of the priors defined on the basis of these effect size estimates were calculated using the 95% confidence intervals for the effect sizes given in McNickle and Carson (2015) (i.e., corresponding to contrasts between Pre and Post0, Post10, Post20, and Post30, respectively)

To facilitate the generation of standardized effect sizes for the data obtained in the present study, and permit comparison with statistics reported conventionally in the context of null hypothesis testing, analogous paired *t* tests were undertaken. As the motivating hypotheses were directional, i.e., the tACS-TMS and TMS-TMS interventions were predicted to increase the magnitude of the AURC values, the *t*-tests were one tailed. Confidence intervals (95%) for the effect size estimate associated with each *t* test (Cohen’s d) were generated using 10,000 bootstrap samples. With respect to the calculation of d, in accordance with recommendations concerning repeated measures designs (Cumming and Finch 2001), the “standardiser” was the estimate of the standard deviation of the measurements obtained prior to the intervention.

### Supplementary analyses

To characterize potential time-dependent differences between conditions in a manner that might satisfy the curiosity of the reader looking for a more typical presentation, we also generated effect size estimates and confidence intervals for contrasts between the paired TMS-TMS and sham conditions, and between the tACS-TMS and sham conditions. Careful consideration of the analysis model is required in the context of experimental designs in which participants are engaged in different conditions (i.e., of non-invasive brain stimulation) at intervals of 1 week or longer. In such circumstances, inter-session variability of measurement contributes to that of the estimates for the experimental conditions. In a study in which a PAS protocol was administered on five separate occasions at 1-week intervals, intra-individual variations in the level of response to the intervention (i.e., across sessions) were considerable (Kim et al. 2017). It is well established that the intra-day variability of TMS-derived measurements is lower than when measurements are obtained several days apart (Beaulieu et al. 2017; Cavaleri et al. 2017). In the present case, the relatedness (i.e., intraclass correlation) across sessions of the AURC measurement obtained prior to the intervention was relatively modest (ICC(3) = 0.34; 95% CI: 0.02–0.68). For this reason, in undertaking the present analyses, we first subtracted (i.e., independently for each participant) the AURC value obtained prior to the intervention from the AURC calculated for all post-intervention measurements. In applying this step (i.e., separately for each condition), the impact of inter-session variability in the pre-intervention measurements was mitigated to some extent. Confidence intervals (95%) for the effect size estimates were generated using 10,000 bootstrap samples. With respect to the calculation of d, in accordance with recommendations concerning repeated measures designs (Cumming and Finch 2001), the “standardiser” was in this case the estimate of the standard deviation of the measurements obtained for the sham condition.

## Results

**Fig. 3:**
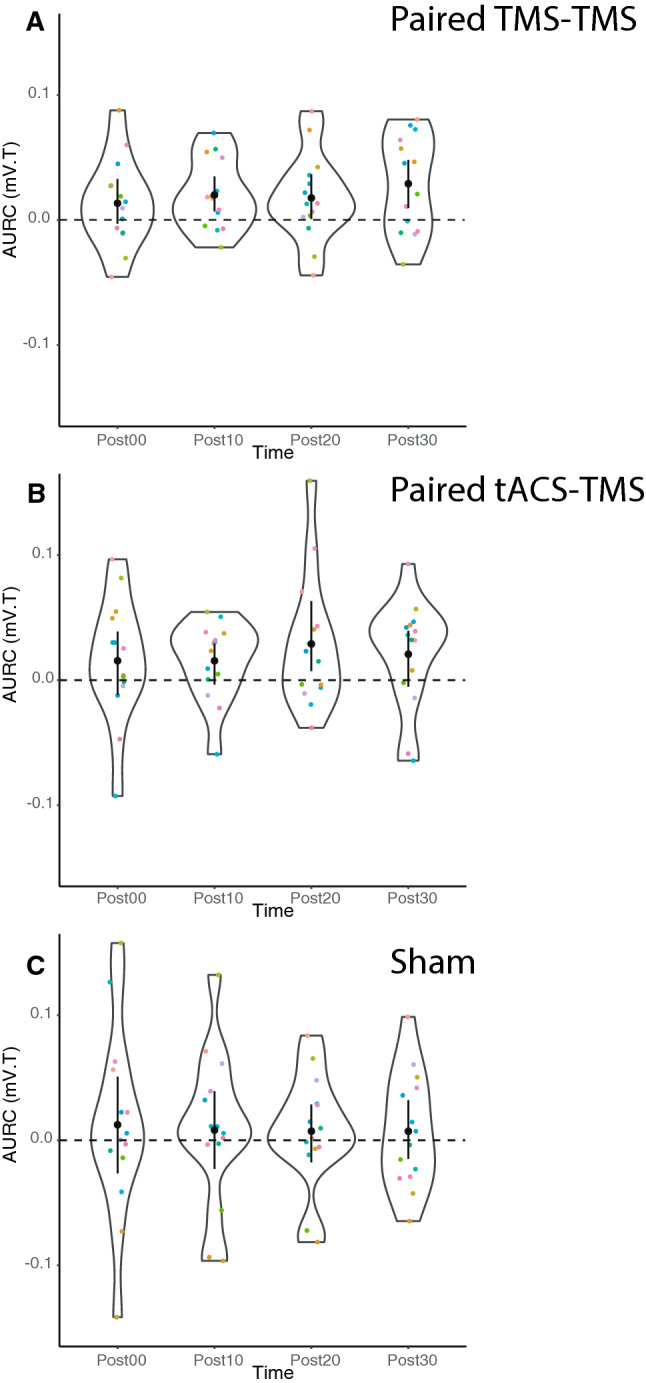
The differences between the AURC values obtained prior to the intervention (Pre), and the AURC values calculated for each time point following the intervention (Post0, Post10, Post20, Post30) are illustrated using separate violin plots for each experimental condition: paired TMS-TMS (**a**); paired tACS -TMS (**b**); and Sham (**c**). Violin plots show the probability density of the data at different values, smoothed by a kernel density estimator. Individual data points corresponding to the difference (post–pre) values (ΔAURC (mV.T)) obtained for each of the 14 participants are also plotted. Each participant is represented by a distinct colour. The filled circle symbols indicate the means. The error bars are the associated 95% confidence intervals derived from 10,000 bootstrap samples. In each case the dashed horizontal line corresponds to zero change in the AURC value obtained following the intervention, relative to the AURC value obtained prior to the intervention

The Bayes factor is a likelihood ratio that expresses the degree to which the data support the experimental hypothesis over the null. If the Bayes factor is greater than 1, the data support the experimental hypothesis over the null. If the Bayes factor is less than 1, the data support the null over the experimental hypothesis. Strictly, the Bayes factor indicates the degree to which the prior odds in favour of an experimental hypothesis (over the null hypothesis) should be multiplied in light of the data (e.g., Dienes 2008). In the present study, each experimental hypothesis was defined quantitatively—in terms of an effect size range, on the basis of previous research (Table 1). It is considered both a strength and weakness of the Bayesian approach that each person can have their own sense of how the odds represented by a Bayes factor should be interpreted. Various guidelines have been written with the intent that Bayes Factors can be comprehended in a scientific context. For example, Wetzels and Wagenmakers (2012) propose that Bayes factors in the range 1–3 be considered “anecdotal evidence”, in the range 3–10 “substantial evidence”, and in the range 10–30 “strong evidence”. Ultimately however it is for the reader to decide how to view the odds in favour of the experimental hypotheses that are reported (Table 2; Fig. 3).

**Table 2:** Bayes Factors (BF) for planned contrasts conducted (separately for each condition) between the AURC value obtained prior to the intervention (Pre), and the AURC value calculated for each time point following the intervention (Post0, Post10, Post20, Post30)

	BF	*t*(13)	*p*	*d*	*d* (lower)	*d* (upper)
TMS-TMS						
Pre vs. Post00	2.0	1.43	0.088	0.43	0.03	1.16
Pre vs. Post10	17.9	2.69	0.009	0.64	0.21	1.05
Pre vs. Post20	3.5	1.88	0.041	0.57	0.07	1.37
Pre vs. Post30	22.5	2.82	0.007	0.93	0.26	1.76
tACS-TMS						
Pre vs. Post00	1.2	1.18	0.130	0.35	0.01	0.98
Pre vs. Post10	3.9	1.85	0.043	0.35	0.01	0.78
Pre vs. Post20	4.9	2.03	0.031	0.66	0.13	1.74
Pre vs. Post30	2.2	1.79	0.049	0.47	0.02	1.08
Sham						
Pre vs. Post00	0.4	0.61	0.276	0.19	0.00	0.83
Pre vs. Post10	0.2	0.49	0.317	0.12	0.00	0.50
Pre vs. Post20	0.2	0.59	0.282	0.11	0.00	0.39
Pre vs. Post30	0.1	0.58	0.284	0.11	0.00	0.44

The test statistic (*t*) and *p* value (*p*) for the corresponding *t*-test are given, along with the associated effect size estimate (d), and the lower and upper bootstrapped 95% bias-corrected and accelerated (BCa) confidence intervals for each effect size estimate

In the paired TMS-TMS condition, the index of corticospinal excitability (AURC) was elevated at 10, 20 and 30 min following the cessation of stimulation (Fig. 3a). The corresponding Bayes factors were 17.9, 3.5, and 22.5 respectively. In other words, 30 min following the cessation of the intervention, the odds in favour of the experimental hypothesis that the effect size (d) corresponding to the (positive) change in corticospinal excitability was in the range 0.634 to 1.063, over the alternative hypothesis that the effect size did not lie within this range, was 22:1.

In the tACS-TMS condition (Fig. 3b), post-intervention elevations in the AURC were less marked. With respect to the Post20 contrast, the odds in favour of the hypothesis that the effect size (d) was in the range 0.556–0.976 (over the alternative hypothesis that it lay outside this range) were approximately 5:1. The calculated effect size was 0.66 (“medium” i.e. > 0.50 (Cohen 1988)). For the Post10 contrast, the odds (with respect to the effect size range 0.474–0.893) were approximately 4:1. In this case, the observed effect size was 0.47.

In the sham condition (Fig. 3c), all Bayes factors were less than 1 (0.1–0.4). The corresponding effect sizes were all less than 0.20.

### Supplementary analyses

In a manner that was consistent with the outcomes of the analyses conducted for the individual conditions, the magnitude of the difference between the paired TMS-TMS and the sham condition was greatest for the Post30 contrast, and the magnitude of the difference between the tACS-TMS and the sham condition was greatest for the Post20 contrast (Table 3). As might be anticipated given the contribution of variability in measurement across testing sessions, the effect sizes obtained for the contrasts between conditions/sessions were smaller than those present for contrasts within sessions. For example, the effect sizes corresponding to the Post30 contrast (versus sham) for the paired TMS-TMS condition (0.48), and to the Post20 contrast (versus sham) for the tACS-TMS condition (0.48), were below the lower boundary of the customary designation of a medium sized effect (0.50).

**Table 3:** Effect size estimates (d) for contrasts between the AURC values obtained in the sham condition and 1) the AURC values obtained in the TMS-TMS condition, and 2) the AURC values obtained in the tACS-TMS condition, respectively, at each time point following the intervention (Post0, Post10, Post20, Post30)

	*d*	*d* (lower)	*d* (upper)
TMS-TMS			
Post00	0.02	< 0.01	0.02
Post10	0.20	< 0.01	0.66
Post20	0.23	< 0.01	0.76
Post30	0.48	0.02	1.24
tACS-TMS			
Post00	0.04	< 0.01	0.13
Post10	0.12	< 0.01	0.42
Post20	0.48	0.03	0.97
Post30	0.30	< 0.01	0.93

The lower and upper bootstrapped 95% bias-corrected and accelerated (BCa) confidence intervals for each effect size estimate are also given

## Discussion

Using Bayes factors to express the degree to which the data supported specific experimental hypotheses—which were defined quantitatively in terms of predicted effect sizes, we demonstrated that two novel forms of associative stimulation increase the excitability of corticospinal projections to the forearm. The weight of evidence in support of the experimental hypotheses was however greater for the variant in which a weak magnetic stimulus was paired repeatedly with a suprathreshold magnetic stimulus delivered to the opposite primary motor cortex, than for the variant in which the first stimulus of the pair was a 500 ms duration burst of (140 Hz) tACS.

The effect sizes obtained empirically were generally smaller for the tACS-TMS condition than for the TMS-TMS condition. One key aspect of Bayesian analyses is that there need not be concordance between the magnitude of the effect size and the magnitude of the associated Bayes factor. A key consideration in this regard is that the prior defines a specific range of effect sizes. In the event that the effect size obtained empirically is, for example, larger than this range, the Bayes factor may be smaller than for a value of the effect size that lies within the pre-specified range. The Bayesian approach thus places an emphasis on precision in the formulation of experimental hypotheses. There is an obvious contrast with NHST that makes a two-way distinction: evidence against the null hypothesis versus anything else. Bayes factors provide for a continuous estimate of the evidence in favour of one model or set of parameters as opposed to another (e.g., Rouder et al. 2009). In this vein it should be noted that in the present case the informed prior defined in conjunction with the generation of Bayes factors were derived from a previous study in which a paired TMS protocol was employed (i.e., Rizzo et al. 2009). The effect sizes obtained empirically for the tACS-TMS condition tended to lie at the lower end of the ranges defined for the experimental hypotheses (i.e., for the individual pre versus post contrasts). In the event that the study was to be repeated, the experimental hypothesis associated with the tACS-TMS Post20 contrast—as an example, would be adjusted (downward) to be defined as the effect size range 0.45–0.87 (using the same confidence intervals), rather than 0.556–0.976 (Table 1). In the event that the same effect size (0.66) was obtained again (and all other factors being equal), the corresponding Bayes factor would be larger.

It has been noted previously for projections to forearm muscles that the magnitude of the increases in corticospinal excitability brought about by an associative protocol (in young adults) tends to increase over a period of time following the cessation of the intervention (e.g.,Carson et al. 2013; McNickle and Carson 2015; Carson and Rankin 2018). This was also the case in the present study. In the paired tACS-TMS condition, the largest AURC values were obtained 20 min after the intervention ended. Following paired TMS-TMS, the largest AURC values were present 30 min later (as per Rizzo et al. 2009, 2011). Indeed, the variation in the magnitude of the effects observed by Rizzo and colleagues over the interval following the associative stimulation was incorporated explicitly in the formulation of the priors used in the present study. This illustrates further that in the deployment of Bayesian analyses, emphasis may be placed on the precise specification of experimental hypotheses, guided either by prior empirical findings or by predictions generated through other means.

## Conclusions

The outcomes of this study emphasise that the induction of increases in the excitability of corticospinal projections arising from a paired associative protocol, does not depend on temporally discrete cortical stimulus events. In the present case a 500 ms burst of 140 Hz 1 mA tACS—when used as the first stimulus in the pair was effective, as was 55% rMT TMS, in promoting subsequent increases in corticospinal excitability. These results contribute to an accumulating body of evidence that associative effects may be expressed at the systems level in humans when the timing of the contributory elements is not precisely circumscribed (e.g.,McNickle and Carson 2015; Shulga et al. 2016). They further support the view that multiple cellular pathways—extending beyond the domain of STDP, are likely to mediate the LTP-type response typically ascribed to PAS (Carson and Buick 2019; Carson and Kennedy 2013; Seeman et al. 2017).

## Data Availability

The data reported in this study are available via: https://zenodo.org. The Digital Object Identifier (DOI) is: 10.5281/zenodo.4010860.
